# The Positive Correlations between the Expression of Histopathological Ubiquitin-Conjugating Enzyme 2O Staining and Prostate Cancer Advancement

**DOI:** 10.3390/ph14080778

**Published:** 2021-08-08

**Authors:** Jae-Heon Kim, Hee-Jo Yang, Chang-Ho Lee, Youn-Soo Jeon, Jae-Joon Park, Kwang-Woo Lee, Jae-Ho Kim, Su-Yeon Park, Su-Jung Song, Yon-Hee Kim, Ah-Rim Moon, Ji-Hye Lee, Yun-Seob Song

**Affiliations:** 1 Department of Urology, Soonchunhyang University School of Medicine, Seoul 04404, Korea; piacekjh@schmc.ac.kr (J.-H.K.); 115803@schmc.ac.kr (J.-J.P.); 2 Department of Microbiology, Soonchunhyang University School of Medicine, Seoul 04404, Korea; 3 Department of Urology, Soonchunhyang University School of Medicine, Cheonan 31151, Korea; c78154@schmc.ac.kr (H.-J.Y.); leech@schmc.ac.kr (C.-H.L.); ysurol@schmc.ac.kr (Y.-S.J.); 4 Department of Urology, Soonchunhyang University School of Medicine, Bucheon 14584, Korea; urolkw@schmc.ac.kr; 5 Department of Urology, Soonchunhyang University School of Medicine, Gumi 39371, Korea; uroseven@schmc.ac.kr; 6 Department of Data Innovation, Soonchunhyang University Seoul Hospital, Seoul 04404, Korea; suyeon1002@schmc.ac.kr; 7 Department of Applied Statistics, Chung-Ang University, Seoul 06974, Korea; 8 Soonchunhyang Institute of Medi-bio Science, Soonchunhyang University, Cheonan 31151, Korea; ssong1@sch.ac.kr; 9 Department of Integrated Biomedical Science, Soonchunhyang University, Cheonan 31151, Korea; 10 Department of Pathology, Soonchunhyang University School of Medicine, Seoul 04404, Korea; yonheenara@schmc.ac.kr; 11 Department of Pathology, Soonchunhyang University School of Medicine, Bucheon 14584, Korea; armoon@schmc.ac.kr; 12 Department of Pathology, Soonchunhyang University School of Medicine, Cheonan 31151, Korea

**Keywords:** UBE2O, ubiquitin, prostate cancer

## Abstract

Background: The mTOR signaling pathway is inactivated by AMPK’s tumor-suppressing function. It is recognized that ubiquitin conjugating enzyme 2O (UBE2O), which directly targets AMPK for ubiquitination and degradation, is intensified in human cancers. Methods: This study investigated the clinical data about prostate cancer. Examination was also carried out into tissue microarrays (TMA) of human prostate cancer (*n* = 382) and adjacent non-neoplastic tissues around prostate cancer (*n* = 61). The TMA slides were incubated with antibodies against UBE2O, and the cores were scored by the pathologist blind to cancer results. Results: Very strong positive correlations were identified between the expression of UBE2O staining and high PSA and pathological stage of prostate cancer. Cox’s proportional hazard analysis established correlations between the following: (1) positive surgical margin and biochemical recurrence free survival, (2) PSA grade and clinical recurrence free survival, (3) regional lymph node positive and clinical recurrence free survival, (4) adjuvant treatment and overall survival, and (5) pathological T stage and overall survival. Conclusion: There is a positive correlation between the expression of UBE2O staining and prognosis for prostate cancer. Thus, a prostate cancer prognosis can be assessed with the expression of UBE2O staining.

## 1. Introduction

In eukaryotes, the ubiquitin proteasome system (UPS) targets cell cycle regulators for proteasome-mediated degradation, thereby strictly controlling the cell cycle at major checkpoints. In order to enable ubiquitination of target proteins, the UPS necessitates the ubiquitin-activating enzyme (E1), the ubiquitin-conjugating enzyme (E2), and the ubiquitin ligases (E3) to work harmoniously. Mono-ubiquitination controls the ubiquitin-reliant endocytosis, rearrangement of protein complexes, repair of DNA, and transcriptional regulation. Poly-ubiquitination, which is a chain of a minimum of four ubiquitin’s added to an individual lysine (Lys) residue, is necessary for the labelling of target proteins for degradation [1].

In terms of the ubiquitin enzymes, the E1 activates ubiquitin by attaching the molecule to an active site cystine (Cys) and then using a thioester linkage, moves the ubiquitin to the E2 active site Cys. Subsequently, via E3-mediated specificity, the E2 gives the ubiquitin from its Cys to Lys of the target protein. The E3 enzyme binds to the target protein that will be degraded [1]. E3 enzymes are part of the domain group that includes the anaphase-promoting complex (also referred to as cyclosome, APC/C) [2]. As their deregulation is linked with cancer, the majority of studies have primarily addressed E3s. It is only of late that the critical part E2s play in the regulation of cell cycle progression and in specific cancer development and progression has begun to receive more attention.

AMP-activated protein kinase (AMPK) is a key monitor of cellular energy and nutrient levels. Associations have been identified between cancer and AMPK loss or activity deregulation. More specifically, it has been established that decreased AMPK activity is found in human breast and kidney cancers [3,4]. It is recognised that UBE2O, which directly targets AMPK for ubiquitination and degradation, is intensified in human cancers. UBE20 is a comparatively large E2 ubiquitin-conjugation enzyme. It is magnified in a subset of human cancers [5,6,7,8,9,10]; however, its contribution in tumorigenesis remains partially undefined. Overexpressed in numerous human cancers, UBE2O targets AMPK for ubiquitination and degradation, which subsequently fosters the activation of the mTOR-HIF1a pathway. Tumorigenesis is impeded by the genetic deletion of UBE2O via the regeneration of AMPKa2 [11].

In UBE2O-deficient TRAMP mice, it was found that there is reduced formations of invasive prostate carcinoma and metastasis [11]. Obstructing UBE2O treatment resulted in decreased prostate lobe enlargement and high G-PIN prostate cancer development in TRAMP in UBE2O positive mice. Additionally, the hindering of UBE2O decreases tumorigenesis to degrees in line with UBE2O deficiency cases [11]. UBE2O plays a key role in the initiation, progression, invasion, and metastasis of prostate cancer. In mouse models of breast and prostate cancers, decreased tumor growth and metastasis rates were observed when one or both UBE2O alleles were lost [11]. The purpose of this study is to examine the relationships between the expression of immunohistochemical UBE2O staining and the progression factors in prostate cancer patients.

## 2. Results

### 2.1. Clinicopathological Characteristics of Prostatic Cancer Patients

Total TMA (*n* = 382) was comprised of 202 prostate cancer patients from Soonchunhyang University Hospital and 180 purchased externally; the surgical margin could be assessed in only the 200 prostate cancer patients from Soonchunhyang University Hospital out of the total 382 patients (Table 1).

### 2.2. UBE2O Expression 

**Figure 1:**
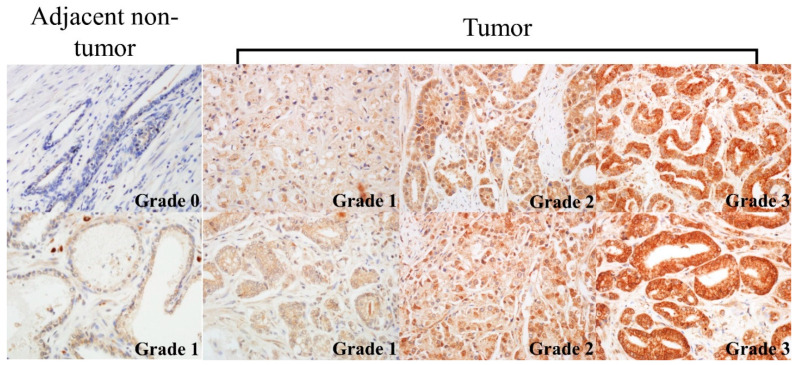
Different expression of UBE20 in non-neoplastic and neoplastic tissues. UBE2O grade—0: negative, 1: weak 2: moderate, 3: strong. (×400).

**Figure 2:**
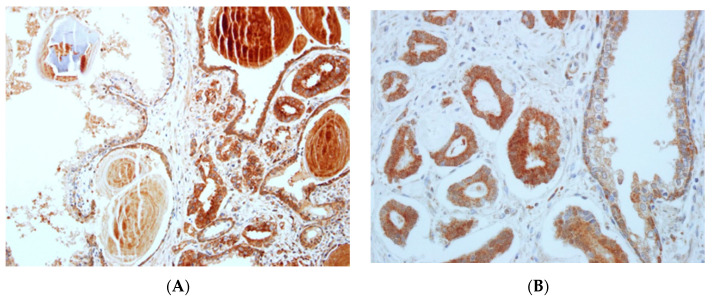
Different expression of UBE20 in neoplastic tissue and non-neoplastic tissue. (**A**) Expression score of UBE20 in right side (neoplastic tissue) was 3 and was 1 in left side (non-neoplastic tissue) with preservation of basal cell. (×200) (**B**) Expression score of UBE20 in left side (non-neoplastic tissue) was 3 and was 1 in right side (neoplastic tissue) with preservation of basal cell (×400).

UBE2O was expressed in cytoplasm of the luminal epithelial cell in the non-neoplastic prostatic tissue. UBE2O in prostatic cancer primarily materialized in the cytoplasm of tumor cells (Figure 1). The classifications for the 355 prostate cancer patients’ expression of UBE2O were as follows: grade 0 (*n* = 0), grade 1 (*n* = 120), grade 2 (*n* = 154), and grade 3 (*n* = 81). Additionally, for the 61 non-neoplastic prostate tissue, expressed UBE2O were as follows: grade 0 (*n* = 22), grade 1 (*n* = 15), grade 2 (*n* = 23), and grade 3 (*n* = 1). Furthermore, prostate cancer tissues and non-neoplastic prostate tissues showed higher and lower grades of UBE2O, respectively (Table 2, Figure 1, Figure 2, Suppl. Figure S1) (*p* < 0.001).

### 2.3. Association of UBE2O Expression with Clinicopathologic Parameters

As mentioned, of the total 382 study population, the UBE2O immunohistochemical staining of prostate cancer could be assessed in 200 patients. It was only possible to assess the surgical margin in 200 patients from Soonchunhyang University Hospital. Of those 200, 173 could be assessed with UBE2O immunohistochemical staining.

Cox’s proportional hazard analysis was performed on the 200 prostate cancer patients from Soonchunhyang University Hospital, and it was found that patients with a higher grade of UBE2O demonstrated higher PSA than those of lower grade UBE2O (*p* < 0.001). Additionally, patients with higher UBE2O grades demonstrated greater pathologic stage than those with lower UBE2O grades (*p* < 0.001) (Table 3).

However, patients with higher UBE2O grades did not present with greater involvement of seminal vesicle than those with lower UBE2O grades. Patients with higher UBE2O grades demonstrated greater involvement of lymph nodes than those with lower UBE2O grades (*p* < 0.05). Finally, patients with higher UBE2O grades did not demonstrate higher positive surgical margins than those with lower UBE2O grades (Table 3).

### 2.4. Survival Analysis

Of the 335 prostate cancer patients, it was possible to evaluate 173 for survival. Biochemical recurrence, clinical recurrence, and overall survival were described in Table 4. Biochemical recurrence (mean ± standard error (SE)) of UBE2O grade 1, UBE2O grade 2, and UBE2O grade 3 were 7.77 ± 0.66 year, 11.39 ± 0.68 year, and 9.43 ± 0.57 year, respectively. Clinical recurrence (mean ± SE) of UBE2O grade 1, UBE2O grade 2, and UBE2O grade 3 were 10.23 ± 0.51 year, 11.82 ± 0.45 year, and 11.31 ± 0.44 year, respectively. Overall survival (mean ± SE) UBE2O grade 1, UBE2O grade 2, and UBE2O grade 3 were 8.47 ± 0.70 year, 9.65 ± 0.60 year, and 10.63 ± 0.46 year, respectively. 

Cox’s proportional hazard analysis in patients with prostate cancer found the following (Table 5 and Table 6): positive surgical margin correlated with biochemical recurrence free survival (*p* = 0.003); clinical recurrence free survival of prostate cancer correlated with PSA grade (*p* = 0.004); clinical recurrence free survival of prostate cancer correlated with regional lymph node positive (*p* < 0.001). Cox’s proportional hazard modelling in patients with prostate cancer found a correlation between adjuvant treatment (*p* = 0.030) and pathological T stage (*p* = 0.020), and overall survival (OS) of prostate cancer (Table 7).

In addition, Cox’s proportional hazard analysis of UBE2O expression in patients with prostate cancer from Soonchunhyang University Hospital identified a correlation with between UBE2O immunohistochemical staining and the following (see Table 5, Table 6 and Table 7): clinical recurrence free (HR = 0.028[95% CI: 0.008–0.106], HR= 0.023[95% CI: 0.006–0.841], *p* < 0.001); Overall survival (HR = 0.503[95%CI: 0.197–1.289], HR = 0.310[95% CI: 0.112–0.856], *p* < 0.05).

Kaplan–Meier survival analysis was performed and subsequently the log-rank test for UBE2O expression in patients with prostate cancer was run for the purposes of comparison. Results showed that there was no correlation between the grade of UBE2O immunohistochemical staining and prostate cancers’ biochemical recurrence (*p* = 0.45), clinical recurrence (*p* = 0.89), or OS (*p* = 0.24). 

## 3. Discussion

In this study, it was found that adjacent non-neoplastic tissues around prostate cancer and prostate cancer itself showed lower and higher grades of UBE2O, respectively (*p* < 0.05). This result signified that UBE2O expression enables distinction between benign and malignant prostate tumors, as immunohistochemical studies in prostate cancer display a substantial disparity of UBE2O staining cells between benign and malignant lesions. The results of this study underscore the importance of the ubiquitination process in the prostate carcinogenesis and in the propagation of prostate cancer cells.

One AMPK tumor-suppressive functions is hindering the synthesis of most cellular macromolecules by deactivating the mTOR signalling pathway [12]. Other functions include the downregulation of the glycolytic pathway to impose an anti-Warburg impact [13,14], halt the cell cycle in conjunction with the stabilisation of p53 and p27Kip1 [15], and combat the epithelial–mesenchymal transition related to tumor invasion and metastasis [16].

Per cBioPortal’s (www.cbioportal.org (accessed on 4 April 2021)) TCGA datasets, UBE2O is upregulated in human breast, bladder, liver, and lung carcinomas. UBE2O expression is amplified and relates to AMPKa2/mTOR/HIF1a in human cancers, using the existing microarray database: Liu’s BCa, breast carcinoma (GEO: GSE22820, *n* = 176) [17]; Stephenson’s CaP, prostate carcinoma (*n* = 97) [8]; Taylor’s CaP (GEO: GSE21032, *n* = 179) [18].

Past studies of microarray-based gene expression have identified a high UBE2O expression rate in a variety of human cancer subsets [11]. Furthermore, immunohistochemical analysis of breast cancer samples determined high UBE20 expression [11]. Another database [19] analyzing cancer patient survival rates shows the clinical significance of UBE2O overexpression. In a substantial proportion of human cancers, UBE2O expression could impact both neoplastic malignancies and clinical outcomes. As UBE2O is upregulated, it may play a role in the regulation of the AMPKa2-mTOR-HIF1a pathway.

In mouse models of breast and prostate cancers, deferred tumor initiation and reduced tumor growth and metastasis rates were found to be attributable to the loss of one or both UBE2O alleles [11]. In cancer cells, UBE2O positively regulates aerobic glycolysis and cellular biosynthesis; the deactivation of UBE2O can switch off the tumor cells’ glycolytic and biosynthetic programs. Therefore, UBE2O can function as an oncogene that initiates cancer progression and removes important metabolic checkpoints that provoke pro-growth cellular metabolism [11]. In cancer, the presence of UBE2O causes upregulation in the mTOR-HIF1a pathway, which is strongly correlated with pro-growth, glycolytic, and biosynthetic programs [11]. In this study, a higher PSA was demonstrated in patients with a higher UBE2O grade (*p* < 0.001). A higher pathologic stage was identified in patients with a higher UBE2O grade (*p* < 0.001). The expression of UBE2O immunohistochemical staining facilitates prostate cancer prognosis.

Furthermore, in mouse cancer models, the removal of UBE2O weakened the intra-tumoral vascularization and expression of neovascularization genes, including HIF1a targets. This underscores the importance of UBE2O’s regulation of the mTOR-HIF1a pathway for both the angiogenic signaling pathway, and the neovascularization necessary for cancer growth and metastasis [11]. It has been found that drugs regulating UBE2O activity function as a form of cancer therapy, as the obstructing of UBE2O with ATO has an impact on tumor biology comparable to that of UBE2O deficiency Human Tumor TMA Analysis [11].

In this study, it was found that patients with higher UBE2O grades did not demonstrate greater seminal vesicle involvement than the lower UBE2O grade patients. Additionally, patients with higher UBE2O grades presented greater lymph node involvement than the UBE2O lower grade (*p* < 0.05). With prostate cancer, lymphatic metastasis can be anticipated with the expression of UBE2O immunohistochemical staining.

In human breast cancer, tumor tissue microarrays (TMAs) presented a high expression of UBE2O staining [11]. UBE2O is often intensified or mutated in many cancers, and its high expression is correlated with low survival rates of breast, gastric, lung, and prostate cancers. In this study, Cox’s proportional hazard analysis ascertained the following correlations: between positive surgical margin and biochemical recurrence free survival; between PSA grade and clinical recurrence free survival between regional lymph node positive and clinical recurrence free survival; between adjuvant treatment and overall survival; between pathological T stage and overall survival. Cox’s proportional hazard modelling for UBE2O expression also identified that UBE2O immunohistochemical staining correlated with clinical recurrence free survival (*p* < 0.001) and overall survival (*p* < 0.05) in prostate cancers (Table 6 and Table 7).

However, the Kaplan–Meier test and the subsequent log-rank comparison test establish that there was no correlation between the grade of UBE2O immunohistochemical staining and prostate cancers’ biochemical recurrence, clinical recurrence, or OS. An assumption is made that in this study, the number of prostate cancer patients was low, as was the prostate cancer mortality rate; thus, there were minimal cancer deaths within the study’s duration. Hence, the expression of UBE2O immunohistochemical staining, PSA, Gleason score, and pathological stage can all predict prostate cancer prognosis.

Although we have explored pilot UBE20 expression using human TMA prostate cancer tissue, UBE2O/antibody used validated antibody from Sigma-Aldrich but could not detect UBE2O expression in any of the 494 prostate cancer tissues. This could be due to the difference of antibody characteristics, however, needs validation study using large human tissue. Recently, a study using the same UBE2O antibody (GTX108039) as we used in mice model was conducted. Vila IK, et al. generated a UBE2O knockout mouse line that has been cross-bred in two transgenic mouse models of spontaneous cancer (transgenic adenocarcinoma mouse prostate (TRAMP) for prostate cancer) [11]. In their result, it has been shown that high expression of UBE2O promotes tumor initiation in mouse models of prostate cancers. The rates of distant metastasis of UBE2O-deficient mice were much lower than those of UBE2O-proficient mice of prostate cancers [11].

## 4. Materials and Methods

### 4.1. Patients and Specimen

A group of 202 patients with prostatic adenocarcinoma diagnoses were recruited. All diagnoses were confirmed both histologically and immunohistochemically, and surgical resection had been performed on each of the 202 patients between January 2002 and December 2012 in the Soonchunhyang University Hospital. Preparation of tumor tissues was conducted via formalin-fixed and paraffin-embedded processing. In order to determine the representative area, hematoxylin-eosin (H&E) slides were retrospectively reassessed by two skilled pathologists. Pathological reports and other medical records were also reviewed to gather clinicopathological information. The criteria from the International Union Against Cancer and World Health Organizations/International Society of Urological Pathology was employed to establish the tumor stage and Gleason score. Following the radical prostatectomy (RP), patient follow-ups were carried out via regular measurement of the serum PSA. Average follow-up time was 132 months (range 1–252). Adjuvant treatment was defined as a case of hormonal or radiation therapy without an increase in PSA within 6 months after surgery.

We graded PSA and Gleason scores according to the American Joint Committee on Cancer Eighth Edition Cancer Staging system. PSA is classified as low risk when <10 ng/mL and high risk when >20 ng/mL [20]. The Gleason score was divided into three categories: low, intermediate, and high grade. Prostate cancers with a Gleason score of 6 or less may be called well-differentiated or low-grade and Gleason score of 7 may be called moderately-differentiated or intermediate-grade. Prostate cancers with Gleason scores of 8 to 10 may be called poorly-differentiated or high-grade [20]. Clinically, stage T2 or less is an organ confined disease and above T3 is classified as locally advanced disease in prostatic cancer. Therefore, we classified the T stage into two grades.

Local scientific ethics committees approved this study (Seoul hospital: 2017-02-002, Bucheon hospital: 2017-03-004, Cheonan hospital: 2017-03-031-024, Gumi hospital: 2017-03-031-002). Other factors evaluated were age, Gleason score, seminal vesicle invasion, lymph node invasion, plasm PSA level, and stage.

### 4.2. Construction of Tissue Microarray (TMA)

As mentioned above, tissue microarrays were built from formalin-fixed paraffin-embedded blocks. The H&E slides were meticulously examined under light microscopy in order to obtain the most representative viable tumor portions (*n* = 202). A 3-mm-diameter cylinder was used to core the corresponding areas of each paraffin block twice, and they were subsequently transferred to a recipient paraffin block via a trephine apparatus (Superbiochips Laboratories, Seoul, Korea). Also included were control or adjacent non-neoplastic tissues (*n* = 39). For tissue validation purposes, one section of the block was stained with H&E.

### 4.3. Purchased Additional Tissue Microarray

There was a shortage of patients with prostate cancer with more than T3 pathological stage in the hospital, therefore TMA containing human prostate cancer (*n* = 180) and adjacent non-neoplastic tissues (*n* = 22) were purchased from AccuMax (ISU ABXIS Co., LTD, Seongnam, Korea). All samples were anonymous, and the pathologist reconfirmed the diagnoses of normal or tumor tissue. The TMA encompassed a range of 180 malignant RP specimens. The two previously mentioned pathologists reassessed the hematoxylin and eosin-stained slides to pinpoint the representative areas. Again, the criteria of the International Union Against Cancer and World Health Organization/International Society of Urological Pathology was applied to establish the tumor stage and Gleason score. As previously, local scientific ethics committees approved the study, and the factors investigated were age, Gleason score, seminal vesicle invasion, lymph node invasion, plasm PSA level, and stage.

### 4.4. Immunohistochemistry and Interpretation

Immunohistochemical analysis of the expression of UBE2O was carried out using anti-UBE2O primary antibodies. Successive 4-μM sections were cut from the TMA tissue blocks. Normal prostatic tissue was selected as positive control. A summary of the process is as follows: the TMA sections were moved to adhesive-coated slides, which were subsequently heated at 60 °C for 60 min to deparaffinize them; the slides were washed in xylene three times; the slides were treated in 5% hydrogen peroxide in methanol at 37 °C for 15 min to prevent endogenous peroxidase activity; the antigens were retrieved through microwave treatment in a pH 6.0 epitope retrieval solution for 20 min; the anti-UBE2O antibody (GTX108039, Genetex, Irvine, CA,, USA) was diluted 1:100 and the sections were incubated overnight with the primary antibody in a humidified chamber at 4 °C; a bond polymer refine detection kit (Leica Biosystem, Wetzlar, Germany) and diaminobenzidine as a chromogen were applied to treat the secondary antibody. Incubations were employed as negative controls (both excluding and preabsorbing the specific antibody), and UBE2O expression was only evaluated on tissue cores that had been well-preserved.

The pathologist scored the TMA cores without any knowledge of the patients’ clinicopathological information. Based on histological scoring, the expression of staining was classified as follows: grade 3—strong (+++); grade 2—moderate (++); grade 1—weak (+); and grade 0—negative. It was determined that 355 of the 382 prostate cancer patients could be successfully stained with UBE2O. Additionally, 61 adjacent non-neoplastic tissues from the 355 patients could be successfully stained with UBE2O.

### 4.5. Statistical Analyses 

Initially, the patients’ baseline variables were evaluated. The correlation between the UBE2O group and the established prognostic factors was examined. Chi-square tests or Fisher’s exact test were employed to analyze the classified data, and results were expressed as *n* (%) in descriptive statistics. Analysis of variance analysis (ANOVA) or the Kruskal–Wallis test were carried out on the continuous data and expressed as mean ± standard error (SE).

It was possible to assess 173 of the 382 prostate cancer patients for survival. Biochemical recurrence was established if the following three criteria were met: (a) a PSA increase of a minimum of 0.2 ng/mL, (b) a minimum of two distinct consecutive measurements, (c) that are a minimum of three months apart. Clinical recurrence was clarified as lesions within the bone observable on a radionuclide bone scan and lymphadenopathy or visceral lesions observable via computed tomography imaging of the abdomen, pelvis, and chest. Patients were deemed at risk from the surgery date until recurrence or until the date of the final PSA test. Patients that were not available for follow-up were cut from the date of their last follow-up or PSA test.

In order to assess the prevalence of each outcome stratified by the UBE2O group, Kaplan–Meier survival analysis was conducted. OS, which is defined as the time from surgery until death (from any cause), was applied. OS curves were plotted based on the Kaplan–Meier approach, and the log-rank test was employed to facilitate comparison. Additionally, in order to evaluate their independent link with OS, Cox’s proportional hazard regression analyses were conducted on UBE2O expression.

Following consideration of patient age at diagnosis, histological grade, pathological stage, PSA category, margin status, and lymph node involvement, Cox’s proportional hazard modelling was carried out in order to evaluate the independent prognostic impact of the UBE2O group. For multivariate analysis, the fixed variable was UBE2O, and other aspects were analyzed via the variable selection method so as to assess the link between the UBE2O variable and the results via Cox’s proportional risk regression analysis. In addition, statistical analysis was performed utilizing SPSS Software (version 26) and Rex (Version 3.5.0, RexSoft Inc., Seoul, Korea), and the statistical significance level was set at *p* < 0.05.

## 5. Conclusions

For prostate cancer, there is a positive correlation between the expression of UBE2O staining and high PSA, pathological stage, and lymph node involvement. Hence, it can be concluded that the expression of UBE2O staining can facilitate the assessment of a prediction for prostate cancer prognosis.

## Figures and Tables

**Table 1:** Clinicopathological properties of patient and UBE2O expression in prostate cancer.

Prognosis Factors	*n* (%) (*n* = 382)
Age (years)	67.0 ± 0.4
PSA (ng/mL)	
<10	220 (57.6%)
10–20	97 (25.4%)
>20	65 (17.0%)
Gleason	
≤6	52 (13.6%)
7	155 (40.6%)
8–10	175 (45.8%)
Pathological stage	
≤T2	162 (42.4%)
≥T3	220 (57.6%)
Seminal vesicle invasion	
Negative	319 (83.5%)
Positive	63 (16.5%)
Lymph node involvement	
Negative	370 (96.9%)
Positive	12 (3.1%)
Surgical margin *	
Negative	115 (57.5%)
Positive	85 (42.5%)
Expression of UBE2O ^#^	
0	0
1	120 (33.8%)
2	154 (43.4%)
3	81 (22.8%)

UBE2O grade—0: negative 1: weak 2: moderate 3: strong. Age was expressed as Mean ± standard error. * The number of surgical margin could be evaluated in only 200 with prostate cancer of our hospital from 382 patients. ^#^ The number of UBE2O immunohistochemical expression could be evaluated in only 355 from 382 patients with prostate cancer of our hospital and purchased TMA.

**Table 2:** UBE2O Immunohistochemical staining of prostate cancer and adjacent non-neoplastic tissues for radical prostatectomy due to prostate cancer.

UBE2O	Adjacent Non-Neoplastic Tissues	Prostate Cancer	Total	*p* Value
0	22	0	22	<0.001
1	15	120	135	
2	23	154	177	
3	1	81	82	
Total	61	355	416	

UBE2O grade—0: negative 1: weak 2: moderate 3: strong. The number of UBE2O immunohistochemical staining of prostate cancer could be evaluated in 416 from patients including 355 prostate cancer tissues and 61 adjacent non-neoplastic tissues around prostate cancer in 382 patients with prostate cancer. 382 patients included 202 patients with prostate cancer of our hospital and 180 from purchased TMA. Adjacent non-neoplastic tissues were 39 from TMA of our hospital, 22 from purchased TMA.

**Table 3:** UBE2O immunohistochemical staining of prostate cancer for radical prostatectomy due to prostate cancer.

	UBE20			Total (*n* = 355)	*p* Value
	1 (*n* = 120)	2 (*n* = 154)	3 (*n* = 81)		
Age	65 (60, 68)	65 (60, 69)	66 (62, 72)	65 (61, 69)	0.029
PSA					<0.001
<10	28	45	39	112	
10–20	92	109	42	243	
>20	0	0	0	0	
Gleason sum					0.168
≤6	17	18	8	43	
7	52	67	25	144	
8–10	51	69	48	168	
Pathological stage					<0.001
≤T2	25	42	39	106	
≥T3	95	112	42	249	
Seminal vesicle invasion					0.772
Negative	101	126	65	292	
Positive	19	27	16	62	
Lymph node involvement					0.015
Negative	120	149	75	344	
Positive	0	5	5	10	
Surgical margin ^#^					0.555
Negative	19	40	35	94	
Positive	12	32	35	79	

UBE2O grade—0: negative, 1: weak, 2: moderate, 3: strong. Age was expressed as Median (lower quadrant, upper quadrant). The number of UBE2O immunohistochemical staining of prostate cancer could be evaluated in 355 from our hospital from 200 patients with prostate cancer of our hospital and 180 from purchased TMA. ^#^ The number of surgical margin could be evaluated in only 200 of our hospital from 382 patients with prostate cancer excluding purchased TMA. Out of 202 patients with prostate cancer of our hospital, the 173 patients could be evaluated with UBE2O immunohistochemical staining.

**Table 4:** Biochemical recurrence free, clinical recurrence free and overall survival time for UBE2O expression—173 from 335 patients with prostate cancer could be evaluated for survival.

UBE2O Grade	Total	Event	Censored	Survival Time			Survival Time		
				Mean ± SE	95% CI		Median ± SE	95% CI	
					Lower	Upper		Lower	Upper
**Biochemical recurrence free survival**									
1	31	5	26	7.77 ± 0.66	6.47	9.06			
2	72	10	62	11.39 ± 0.68	10.07	12.72			
3	70	14	56	9.43 ± 0.57	8.32	10.55			
Total	173	29	144	10.77 ± 0.54	9.71	11.83			
**Clinical recurrence free survival**									
1	31	1	30	10.23 ± 0.51	9.23	11.23			
2	72	5	67	11.82 ± 0.45	10.94	12.70	12.42 ± 3.87	4.84	20.00
3	70	5	65	11.31 ± 0.44	10.45	12.18			
Total	173	11	162	11.74 ± 0.34	11.08	12.40	12.42 ± 2.40	7.72	17.11
**Overall survival**									
1	31	7	24	8.47 ± 0.70	7.10	9.84			
2	72	17	55	9.65 ± 0.60	8.47	10.84	10.97 ± 1.72	7.61	14.33
3	70	11	59	10.63 ± 0.46	9.74	11.53	12.11 ± 3.07	6.09	18.13
Total	173	35	138	10.17 ± 0.39	9.41	10.94	12.11 ± 1.11	9.93	14.29

UBE2O grade—0: negative, 1: weak, 2: moderate, 3: strong.

**Table 5:** Cox proportional hazard modelling of UBE2O after accounting for biochemical recurrence free survival—173 from 335 patients with prostate cancer could be evaluated for survival.

	Univariate				Multivariate			
	HR	Lower	Upper	*p*-Value	HR	Lower	Upper	*p*-Value
Age	0.998	0.946	1.054	0.950				
Height	0.947	0.896	1.001	0.053				
Weight	0.985	0.951	1.020	0.394				
Adjuvant treatment: no	Reference				Reference			
Adjuvant treatment: yes	20.171	7.139	56.993	<0.001	8.765	2.829	27.153	<0.001
UBE2O grade 1	Reference				Reference			
UBE2O grade 2	0.619	0.207	1.854	0.392	0.649	0.203	2.074	0.466
UBE2O grade 3	1.035	0.372	2.883	0.947	0.629	0.216	1.832	0.395
PSA grade 0	Reference							
PSA grade 1	2.170	0.983	4.789	0.055				
PSA grade 2	2.769	1.273	6.022	0.010				
Gleason score grade 0	Reference							
Gleason score grade 1	2.037	0.432	9.617	0.369				
Gleason score grade 2	4.923	1.162	20.86	0.031				
Pathologic T stage grade 0	Reference				Reference			
Pathologic T stage grade 1	3.880	1.974	7.627	<0.001	2.165	0.859	5.459	0.102
Seminal vesicle invasion: negative	Reference							
Seminal vesicle invasion: positive	3.757	1.919	7.357	<0.001				
Regional lymph node: negative	Reference							
Regional lymph node: positive	5.712	2.483	13.142	<0.001				
Surgical margin: negative	Reference				Reference			
Surgical margin: positive	5.987	2.732	13.122	<0.001	5.261	1.737	15.931	0.003

UBE2O grade 0: negative, 1: weak, 2: moderate, 3: strong. PSA grade 0: PSA < 10ng/mL, 1: PSA 10–20 ng/mL, 2: PSA > 20 ng/mL. Gleason score grade—0: Gleason score ≤ 6, 1: Gleason score 7, 2: Gleason score ≥ 8. Pathologic T stage grade—0: ≤T2, 1: ≥T3.

**Table 6:** Cox proportional hazard modelling of UBE2O after accounting for clinical recurrence free survival—173 from 335 patients with prostate cancer could be evaluated for survival.

	Univariate				Multivariate			
	HR	Lower	Upper	*p*-Value	HR	Lower	Upper	*p*-Value
Age	1.074	0.976	1.182	0.142				
Height	0.936	0.854	1.026	0.155	1.276	1.134	1.435	<0.001
Weight	0.933	0.875	0.995	0.036	0.807	0.710	0.918	<0.001
Adjuvant treatment: no	Reference							
Adjuvant treatment: yes	19.624	2.544	151.376	0.004				
UBE2O grade 1	Reference							
UBE2O grade 2	1.356	0.151	12.169	0.786	0.028	0.008	0.106	<0.001
UBE2O grade 3	1.644	0.191	14.150	0.651	0.023	0.006	0.084	<0.001
PSA grade 0	Reference							
PSA grade 1	2.473	0.715	8.546	0.153	6.938	1.847	26.064	0.004
PSA grade 2	1.798	0.429	7.537	0.422	0.213	0.027	1.708	0.145
Gleason score grade 0								
Gleason score grade 1								
Gleason score grade 2								
Pathologic T stage grade 0	Reference							
Pathologic T stage grade 1	4.154	1.272	13.562	0.018				
Seminal vesicle invasion: negative	Reference							
Seminal vesicle invasion: positive	5.392	1.735	16.756	0.004				
Regional lymph node: negative	Reference							
Regional lymph node: positive	12.474	3.933	39.567	<0.001	90.544	22.747	360.410	<0.001
Surgical margin: negative	Reference							
Surgical margin: positive	3.169	0.967	10.381	0.057				

UBE2O grade—0: negative, 1: weak, 2: moderate, 3: strong. PSA grade 0: PSA < 10 ng/mL, 1: PSA 10–20 ng/mL, 2: PSA > 20 ng/mL. Gleason score grade—0: Gleason score ≤ 6, 1: Gleason score 7, 2: Gleason score ≥ 8. Pathologic T stage grade—0: ≤T2, 1: ≥T3.

**Table 7:** Cox proportional hazard modelling of UBE2O after accounting for overall survival—173 from 335 patients with prostate cancer could be evaluated for survival.

	Univariate				Multivariate			
	HR	Lower	Upper	*p*-Value	HR	Lower	Upper	*p*-Value
Height	0.980	0.931	1.031	0.434				
Weight	0.973	0.939	1.009	0.139				
Age	1.032	0.981	1.087	0.225	1.052	0.99	1.117	0.102
Adjuvant treatment: no	Reference				Reference			
Adjuvant treatment: yes	0.733	0.379	1.417	0.356	0.399	0.174	0.915	0.030
UBE2O grade 1	Reference				Reference			
UBE2O grade 2	0.776	0.320	1.883	0.575	0.503	0.197	1.289	0.152
UBE2O grade 3	0.473	0.182	1.230	0.125	0.310	0.112	0.856	0.024
PSA grade 0	Reference							
PSA grade 1	1.332	0.633	2.805	0.451				
PSA grade 2	1.573	0.748	3.309	0.233				
Gleason score grade 0	Reference							
Gleason score grade 1	0.869	0.349	2.159	0.762				
Gleason score grade 2	0.707	0.298	1.678	0.431				
Pathologic T stage grade 0	Reference				Reference			
Pathologic T stage grade 1	1.818	0.981	3.370	0.058	2.458	1.155	5.232	0.020
Seminal vesicle invasion: negative	Reference							
Seminal vesicle invasion: positive	1.915	0.954	3.843	0.067				
Regional lymph node: negative	Reference							
Regional lymph node: positive	2.098	0.817	5.386	0.123				
Surgical margin: negative	Reference							
Surgical margin: positive	1.183	0.638	2.191	0.594				

UBE2O grade—0: negative, 1: weak, 2: moderate, 3: strong. PSA grade 0: PSA < 10 ng/mL, 1: PSA 10–20 ng/mL, 2: PSA > 20 ng/mL. Gleason score grade—0: Gleason score ≤ 6, 1: Gleason score 7, 2: Gleason score ≥ 8. Pathologic T stage grade—0: ≤T2, 1: ≥T3.

## Data Availability

Data is contained within the article and supplementary material.

## Supplementary Materials

The following are available online at https://www.mdpi.com/article/10.3390/ph14080778/s1, Figure S1: Representative photographs of high UBE2O expression in prostate cancer samples and low UBE2O expression in non-cancerous prostate.
