# Supplementary material for: The Positive Correlations between the Expression of Histopathological Ubiquitin-Conjugating Enzyme 2O Staining and Prostate Cancer Advancement
Source: Pharmaceuticals (Basel). 2021 Aug 8;14(8):778. doi: 10.3390/ph14080778 (PMC8398491; doi:10.3390/ph14080778)
Supplement: Supplementary file 1 [file pharmaceuticals-14-00778-s001.zip › pharmaceuticals-1254879-supplementary.pdf]

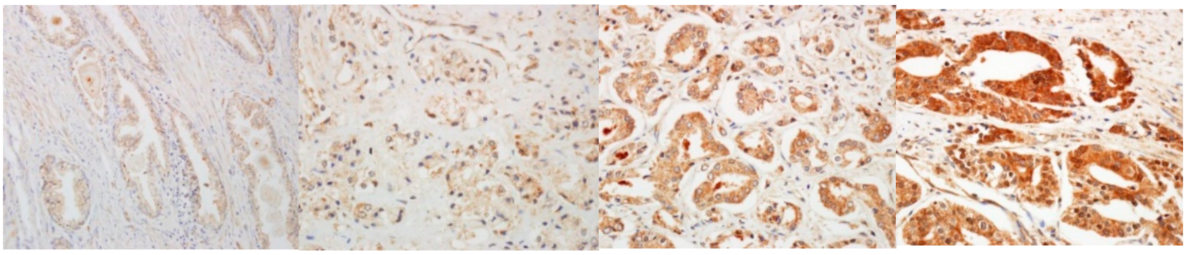

A B C D

Figure S1. Representative photographs of high UBE2O expression in prostate cancer samples and low UBE2O expression in non-cancerous prostate. A: non-cancerous prostate, B: weak, C: moderate, D: strong. Original magnification ×200.
